# Nanoscaled Mechanical Properties of Cement Composites Reinforced with Carbon Nanofibers

**DOI:** 10.3390/ma10060662

**Published:** 2017-06-16

**Authors:** Salim Barbhuiya, PengLoy Chow

**Affiliations:** Department of Civil Engineering, Curtin University, Perth 6845, Australia; chowpengloy2013@yahoo.com.sg

**Keywords:** CNF, nanoindentation, SEM, compressive strength, Young’s modulus

## Abstract

This paper reports the effects of carbon nanofibers (CNFs) on nanoscaled mechanical properties of cement composites. CNFs were added to cement composites at the filler loading of 0.2 wt % (by wt. of cement). Micrographs based on scanning electron microscopy (SEM) show that CNFs are capable of forming strong interfacial bonding with cement matrices. Experimental results using nanoindentation reveal that the addition of CNFs in cement composites increases the proportions of high-density calcium-silicate-hydrate gel (HD-CSH) compared to low-density CSH gel. It was also found that the inclusion of CNFs increases the compressive strength of cement composites.

## 1. Introduction

Plain cement composites suffer from low tensile strength and limited strain capacity. This gives rise to the formation of nano-cracks under relatively low tensile loads. These nano-cracks have a high impact on the durability of cement matrices. Konsta-Gdoutos et al. [1] have shown that the incorporation of carbon nanotubes (CNTs) as the reinforcing material can control the nanoscaled cracks. In particular, the embedded CNTs were found to improve mechanical properties of nano-composites significantly [2,3]. Carbon nanofibers (CNFs) exhibit a similar potential as effective reinforcements in cement composites. This is because they possess excellent material properties such as high stiffness, tensile strength, excellent electrical, and thermal conductivities and corrosion resistance [4,5,6]. Moreover, the physical configuration of CNFs presents a number of exposed edges along the surface, which may establish p areas of interactions with hydration products of cements [7,8].

The reinforcing capabilities of CNFs in cement composites have not been fully realized as done in the case of CNTs. This is mainly due to the challenge in achieving proper fibre to matrix interaction [9]. Nevertheless, most research on cement composites reinforced with CNFs to date has focused on the macroscopic properties but little research has been conducted on the nanoscaled mechanical properties, which are believed to highly impact the macro-scaled properties of materials such as strength, durability, and fracture behavior. In this study, nanoscaled mechanical properties of cement composites reinforced with CNFs were investigated with the aid of nanoindentation technique. Compressive tests were also performed to assess the effects of CNFs on the macroscopic strength of cement composites.

## 2. Experimental Methods

### 2.1. Materials and Mixing

Ordinary Portland cement (OPC) was supplied by Swan Cement, Perth, Western Australia. CNFs used were as-received from US Research Nanomaterials, Inc., Houston, TX, USA, which were produced by chemical vapor deposition (CVD) method. The properties of CNFs used are summarised in Table 1. OPC/CNF composites with 0.2 wt % of cement were investigated, as compared with those neat OPC. The loading of 0.20 wt % is selected based on the existing literature [10,11] to offer the optimization of the properties of nanocomposites. The water–cement ratio used was 0.4 for both cases. Previous research has shown that a polycarboxylate-based superplasticiser effectively dispersed CNTs with minimal effects on the hydration time [12]. Therefore, it was added at 0.10 wt % and 0.40 wt % of cement in OPC and OPC/CNF composites, respectively. The superplasticiser was added to a plastic beaker with water and stirred until a visually mixed solution was obtained. The CNF solution was then ultrasonicated. The mechanical mixing was subsequently performed using a small rotary mixer.

### 2.2. Sample Preparation

The cube samples (50 mm) were prepared for compression test. For the nanoindentation, relatively small cube samples (10 mm) were prepared. All cast samples were kept in a temperature-controlled (20 °C) curing room and demolded after 24 h. After the demolding process, the samples were bath-cured in lime-saturated water and kept in the curing room. The compression tests were performed after 7, 28, and 56 days, respectively. Small flat broken pieces resulting from compression tests were collected for SEM. Samples for nanoindentation were ground and polished using silicon carbide papers with reduced gradations of 52, 35, 22, and 15 μm to expose sample surfaces. Further, samples were impregnated using red-pigmented epoxy resin to provide structural support to fragile porous cement matrices. Once impregnated, samples were put through a final stage of grinding and polishing using reduced carbide papers of 52, 35, 22, and 15 μm, as well as diamond suspensions of reduced gradations of 9, 6, 3, 1, and 0.05 μm on a polishing cloth. Samples were then mounted onto sample disks, further placed into samples trays, and installed into the indenter prior to nanoindentation tests.

### 2.3. Test Methods

#### 2.3.1. SEM

Tescan Mira3 (Brno, Czech Republic) Field Emission Scanning Electron Microscope (SEM) was used to observe the CNF dispersion and interfacial bonding features between CNFs and cement matrices.

#### 2.3.2. Nanoindentation

**Figure 1:**
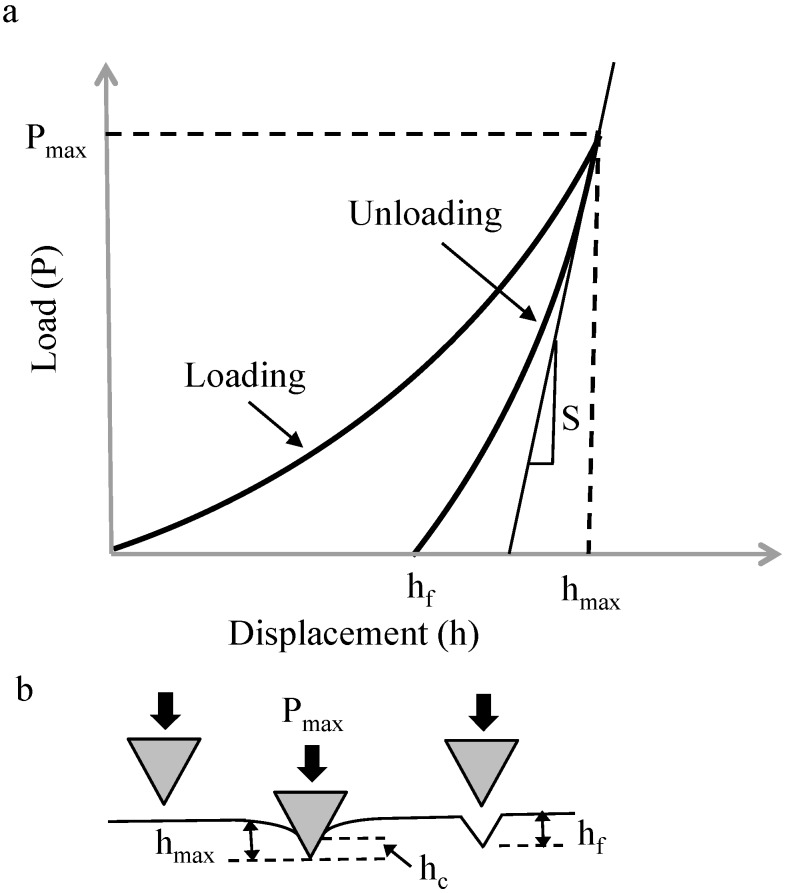
Typical indentation process (**a**) load-displacement curve; (**b**) Interaction between indenter and specimen.

In nanoindentation tests were carried out using Agilent Nano Indenter G200 (Keysight Technologies, Inc., Santa Rosa, CA, USA), In these tests, a controlled load was applied on material surfaces in order to induce local deformation. Using well-established equations based on the principles of elastic contact theory [13], reduced elastic modulus and hardness were calculated. The applied load and the corresponding displacement were continuously monitored during the test. This resulted in a typical load-displacement curve (Figure 1a). The interaction between the indenter tip and the specimen surface during the indentation process is illustrated in Figure 1b. The slope at the beginning of the unloading curve is defined as the contact stiffness (*S*), which is given by

(1) $$S=\frac{dP}{dh}$$

where *P* is the indentation load and *h* is the indentation depth.

The initial part of the unloading curve is fitted by a power law equation

(2) $$S=\frac{2\beta }{\sqrt{\pi }}{\left(\frac{1}{E_{r}}\right)}^{-1}\sqrt{A_{c}}$$

where *E_r_* is the reduced modulus, *A_c_* is the contact area of the indenter, and *β* is a constant for the indenter geometry.

*E_r_* is related to the elastic modulus of the sample (*E*) and the elastic modulus of the indenter (*E_i_*) by the equation

(3) $$\frac{1}{E_{r}}=\frac{1-v^{2}}{E}+\frac{1-v_{i}^{2}}{E_{i}}$$

where *ν* and *ν_i_* are Poisson’s ratios of the sample and the indenter, respectively.

For the Berkovich indenter, the *E_i_* and *ν_i_* are known to be 1140 GPa and 0.07 [14]. Therefore, the reduced elastic modulus, *E_r_* can be defined as

(4) $$E_{r}=\frac{\sqrt{\pi }}{2\beta }\;\frac{S}{\sqrt{A_{c}}}$$

The hardness is defined by

(5) $$H=\frac{P_{\max}}{A_{c}}$$

where *P*_max_ is the peak load.

The nanoindentation machine used in this study was fitted with a Berkovich indenter tip. The calibrated contact area function was derived from indentation tests conducted previously on a fused quartz standard specimen. All tests were programmed in such a way that the loading started when the indenter came into contact with the test surface. The load was maintained for 30 s at the pre-specified maximum value before unloading. The unloading data for the lower indentation depth (i.e., *h_p_* = 300–400 nm) was used to determine the reduced modulus and hardness values of the indentation point. Information on the nanoscaled mechanical properties was obtained from a matrix of 320 indents on the surface of the samples. The selected indent spacing was 20 μm. Each test area was selected by manual inspection using the indenters built in the microscope attached to the nanoindentation machine. The experimental data (i.e., Young’s modulus) were then statistically analyzed to produce a frequency histogram.

#### 2.3.3. Nanoscratch

The nanoscratch tests were carried out using Agilent Nano Indenter G200 (Keysight Technologies, Inc., Santa Rosa, CA, USA) by moving the indenter tip. This was made when the indenter tip was in contact with the specimen surface. The Berkovich tip was used to conduct pre- and post-scratch scans of the surface with a force of 5 μN at a rate of 2 μm/s. Data were recorded during these scans at a rate of 5 points/μm. A scratch length of 100 μm was scratched at each focused site, with the indentation tip used to scan the approach and parting zones of the scratch site at 20 μm each. During the scratching step, a velocity of 2 μm/s was used to apply a maximum load of 50 mN, which is imposed perpendicular to the plane of sample faces.

#### 2.3.4. Compressive Strength Test

The compressive strength was determined in accordance to AS 1012.9-2014 [15]. This was performed using a Multifunctional Control Console (MCC8) machine (Milan, Italy). A total of three specimens were used for the testing at 7, 28, and 56 days. This was calculated from the maximum force applied on the specimen divided by the cross-sectional area of the specimen.

## 3. Results and Discussion

### 3.1. SEM Characterisation

**Figure 2:**
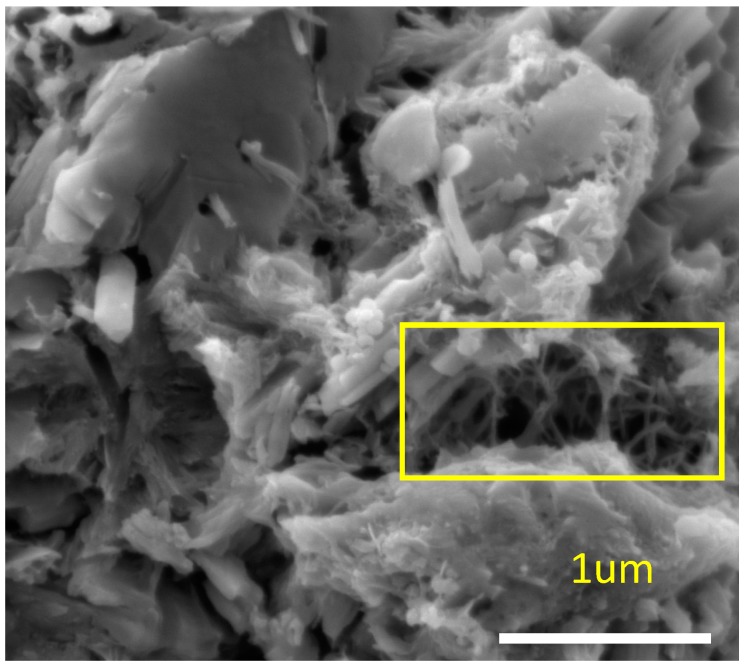
Bridging of cracks by CNFs.

**Figure 3:**
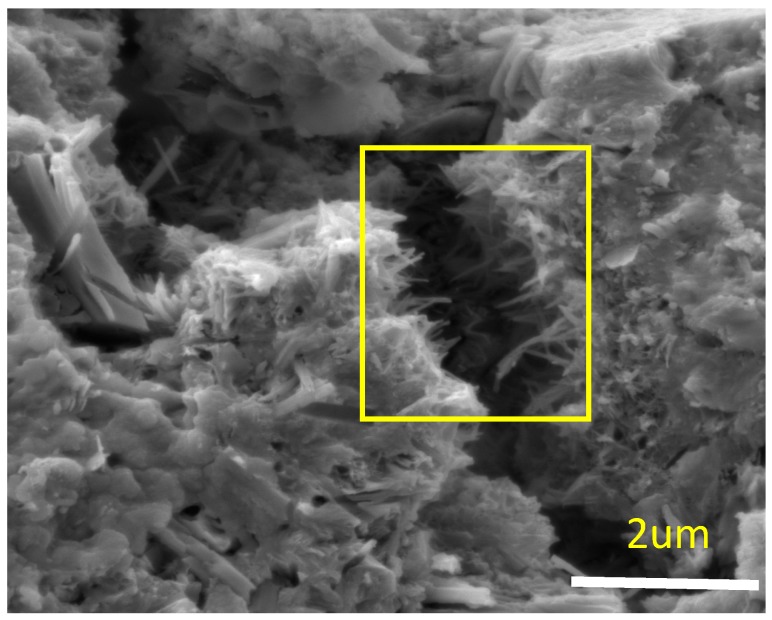
Yielding of CNFs at failure.

The SEM image shown in Figure 2 depicts the bridging of cracks by CNFs. The yielding of CNFs at failure is shown in Figure 3. It can be seen that the individual end strands of the fibers are well entrenched within the surrounding hydration products, which suggests that strong interfacial bonding with good interactions between CNFs and cement matrices has been achieved. The theoretical interpretation can be due to the desirable physical characteristics possessed by CNFs, including exposed edge planes to allow for interfacial interactions with hydration products. The magnification of SEM images does not allow for fiber structures to be viewed, but the practical interactions with hydration products provides clear evidence to support the current study. The observations made are based upon the localized SEM images produced, which are indicative only of a very small portion of the specimens. Hence, a global visualization of fractured morphological structures is required for the future research work. 

### 3.2. Nanoindentation

**Figure 4:**
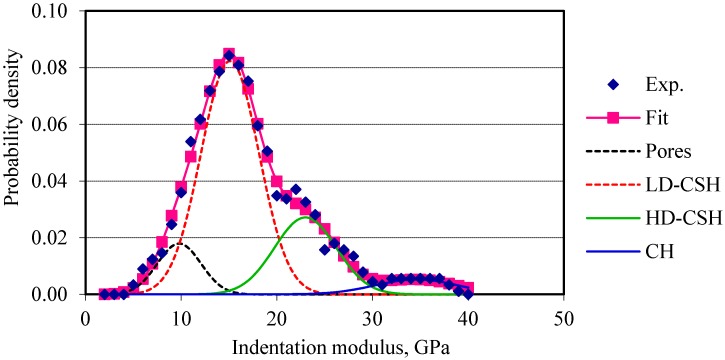
Frequency plot of elastic modulus for OPC.

**Figure 5:**
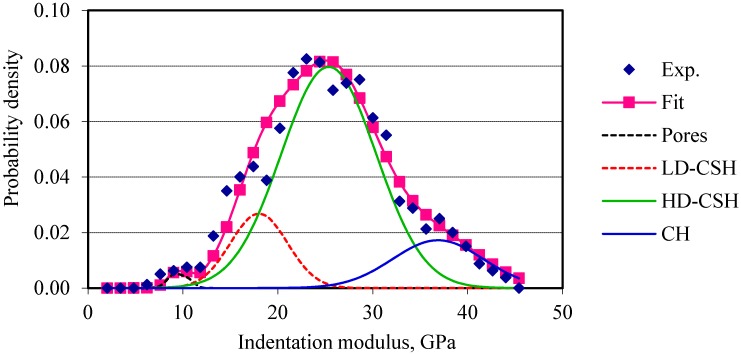
Frequency plot of elastic modulus for OPC/CNF composites.

The indentation moduli are plotted against their probability of occurrences. Four normal distributions corresponding to the four phases (pores, low-density calcium silicate hydrate, high-density calcium silicate hydrate, and calcium hydroxide) are fitted to the probability plots of the elastic modulus of cement composites. For OPC and OPC/CNF composites, nanoindentation data obtained are in good agreement with the data available in the literature, as can be seen in Table 2. Figure 4 and Figure 5 show the frequency plots of elastic modulus for OPC and OPC/CNF composites, respectively. The elastic modulus values greater than 45 GPa are not included in this plot. This is because values greater than 45 GPa can be attributed to the clinker phases. From Figure 4 and Figure 5, it can be seen that, for plain cement composites, the mean peak of the elastic frequency plot falls in the low-density CHS gel region (10–25 GPa). When CNFs were added, the high-density CSH gel region (25–30 GPa) was more pronounced. This indicates an increase in the high-density CSH gel compared to low-density CSH gel.

### 3.3. Nanoscratch

**Figure 6:**
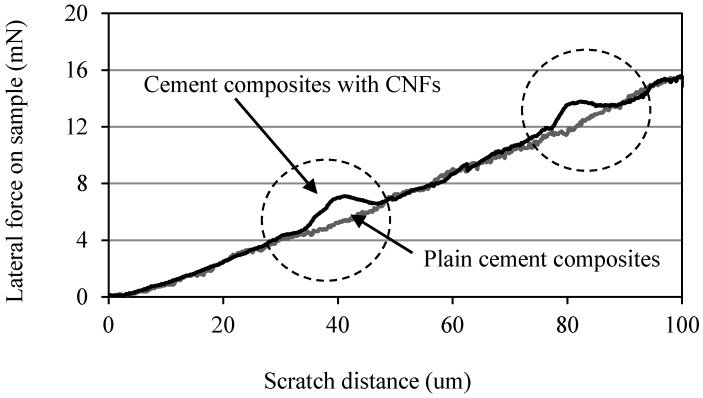
Lateral force required by the indenter in OPC and OPC/CNF composites.

The lateral force required by the indenter to displace the material as a function of the scratch length is shown in Figure 6. It can be seen that, for both OPC and OPC/CNF composites, the lateral force steadily increased from 0 to 15.5 mN in order to displace a scratch length of 100 μm. This contradicts the findings obtained by Lahiri et al. [22], in which it was observed that the lateral force required to displace the material increases in the presence of CNTs. However, in this study, there was a slight variation in the force required to dislocate the material with minor jumps in the lateral force (denoted by circles in Figure 6). These small jumps could be due to the presence of CNFs in the cement matrices. 

**Figure 7:**
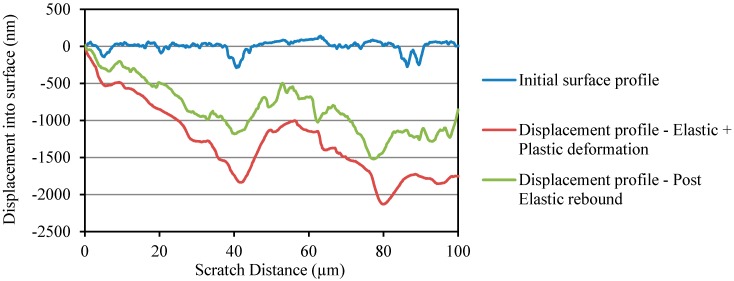
Penetration depth profile for plain OPC.

**Figure 8:**
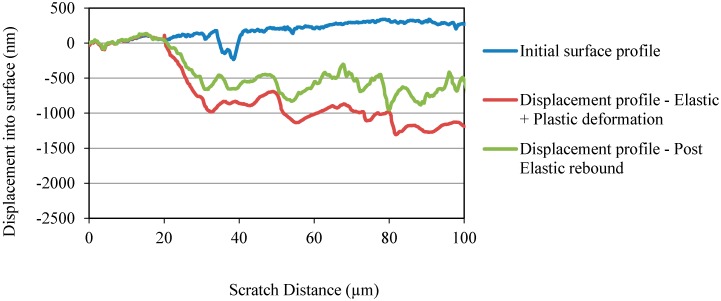
Penetration depth profile for OPC/CNF composites.

Three different scratch profiles corresponding to initial surface profile, displacement profile due to elastic and plastic deformations, and displacement profile due to post elastic rebound are shown in Figure 7 and Figure 8 for OPC and OPC/CNF composites, respectively. From the scratch profiles, it is evident that the indenter penetrated more into the OPC surface compared to OPC/CNF composites. The deepest penetration in OPC/CNF composites was observed to be less than 1275 nm, as opposed to over 2000 nm for OPC. The penetration depth is a mechanical response of the material, which means that a shallow penetration is induced by harder materials. As expected, CNFs as rigid fillers into OPC make their composites harder to go through by the indenter.

### 3.4. Compressive Strength

**Figure 9:**
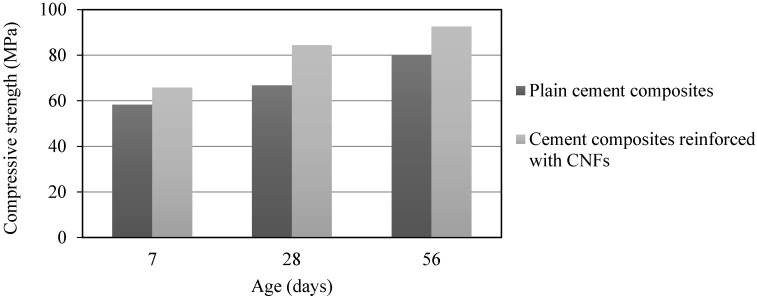
Compressive strength development.

The compressive strengths of OPC and OPC/CNF composites are illustrated in Figure 9. It can be seen at all ages inclusion of CNFs in OPC increased the compressive strength, consistently over those of the OPC. The increase is much higher than that was found by Sanchez and Ince [23], where only a 5% increase was found with the addition of CNFs at 0.5 wt % of cement.

## 4. Conclusions

The nanoscaled mechanical properties of cement composites reinforced with CNFs at 0.2 wt % of cement have been studied as a typical case in this research when compared to OPC. SEM images show that CNFs successfully induce the phenomenon of crack bridging. According to nanoindentation tests, it can be concluded that when CNFs are added in OPC, there tends to be an increase in the high-density CSH gel at the cost of low-density CSH gel. The lateral forces required to displace both OPC and OPC/CNF composites are identical. However, minor jumps were observed in composites. The indenter penetrates more into the OPC surface compared to OPC/CNF composites. The inclusion of CNFs at 0.2 wt % of cement was found to increase the compressive strength of OPC.

## Figures and Tables

**Table 1:** Properties of CNF used.

Parameters	Values
Purity	>99.9%
Outside diameter	200–600 nm
Length	5–50 μm
Specific surface area	18 m^2^/g
Electrical conductivity	>100 s/cm

**Table 2:** Values of elastic moduli from literatures (mean ± SD).

Phase	Elastic Modulus (GPa)	References
Pores	9.1 ± 2.3	[16]
Low-density CSH	21.7 ± 2.2	[17]
	22.5 ± 5.0	[18]
	23.4 ± 3.4	[19]
High-density CSH	29.4 ± 2.4	[17]
	30.4 ± 2.9	[18]
	31.4 ± 2.1	[19]
Calcium hydroxide	36 ± 3.0	[20]
Clinker	125 ± 25	[21]
